# The association between self-compassion and eating disorders among female college students, the mediating role of psychological resilience, and the moderating role of physical exercise

**DOI:** 10.3389/fpsyg.2026.1788344

**Published:** 2026-08-04

**Authors:** Linlin Li, Haijin Pan

**Affiliations:** 1 PE College, Huanghuai University, Zhumadian, Henan, China; 2 Physical Education and Military Teaching Department, Hainan Vocational University of Science and Technology, Haikou, Hainan, China

**Keywords:** eating disorders, female college students, physical exercise, psychological resilience, self-compassion

## Abstract

**Objective:**

Eating disorders among female college students are often accompanied by psychological adjustment difficulties. This study examined the association between self-compassion and eating disorders and investigated the mediating role of psychological resilience and the moderating role of physical exercise.

**Methods:**

A cross-sectional questionnaire survey was conducted among 876 female college students in China. Standardized measures were used to assess self-compassion, psychological resilience, physical exercise, and eating disorders. A moderated mediation model was tested using SPSS.

**Results:**

Self-compassion was negatively associated with eating disorders (β = −11.130, *p* < 0.001). Psychological resilience significantly mediated this association (β = −7.195, *p* < 0.001). Physical exercise significantly moderated the direct association between self-compassion and eating disorders (β = 0.230, *p* < 0.001), with the negative association becoming weaker at higher levels of physical exercise.

**Conclusion:**

Higher self-compassion was associated with lower levels of eating disorders among female college students, and psychological resilience partially accounted for this association. Physical exercise served as a boundary condition for the direct association. These findings highlight the importance of strengthening self-compassion and psychological resilience while considering individual differences in physical exercise.

## 1 Introduction

In recent years, eating disorders have become a growing mental health concern worldwide, and they are particularly prevalent among college students. Female students appear to be more vulnerable to these problems (Eck and Byrd-Bredbenner, 2021; Zheng et al., 2020). Eating disorders often involve persistent and stable patterns of maladaptive eating and weight-control practices, such as extreme dieting, binge episodes, and excessive fixation on physical appearance (Yoon et al., 2023). Recent studies using Chinese college student samples further show that the risk of eating disorders is not uncommon. For example, Wang J. et al. (2023) found that 10.4% of students in a Chinese college student sample showed a risk of eating disorders. Existing epidemiological research also shows that detection rates of anorexia nervosa and bulimia nervosa among Chinese female college students have approached levels reported in Western countries (Wu et al., 2023). This indicates that eating disorders have become a practical concern in the mental health of Chinese college students.

For female college students in China, the risk of eating disorders has specific cultural and developmental contexts. Social expectations about women’s appearance, body management, and self-presentation in Chinese society may make female college students more likely to link body shape and appearance with personal value, social evaluation, and interpersonal attractiveness (Zheng et al., 2020). At the same time, identity exploration during college, peer comparison, idealized body images on social media, and the spread of globalized and Westernized beauty standards may also be associated with stronger body dissatisfaction and greater pressure for weight control (Yoon et al., 2023). Against this background, some female college students may use dieting, excessive exercise, or other weight-control behaviors to cope with appearance-related pressure, and these behaviors may be linked to an increased risk of eating disorders (Ali and Keel, 2023). Related review studies show that eating disorders often co-occur with medical and psychological comorbidities, including abnormal nutritional intake, problems in weight regulation, and symptoms related to depression and anxiety (Kowalewska et al., 2024). Without timely support, these problems may develop into more severe mental disorders and impose long-term burdens on academic adjustment, career development, and interpersonal relationships (Patel et al., 2021). Therefore, it is imperative to further elucidate the psychological factors related to eating disorders among female college students, so that universities can improve risk identification and provide more targeted support strategies.

Although many studies have examined the link between self-compassion (SC) and eating disorders (Shaw and Cassidy, 2022), the specific mechanisms and boundary conditions of this relationship among Chinese female college students still need further clarification. In particular, in contexts where appearance evaluation, body management, and social comparison pressure are closely linked, merely explaining the general association between SC and eating disorders is not enough to clarify how different psychological and behavioral resources are jointly related to eating disorders. Conservation of resources (COR) theory proposes that people manage stress and challenges by gaining, protecting, and restoring resources (Hobfoll, 1989). From this perspective, SC can be understood as an internal resource that is associated with less self-criticism and fewer negative emotional responses related to the body (Fergerson and Brausch, 2022; Voon et al., 2021). Psychological resilience is an adaptive resource that may help individuals maintain a more stable state of psychological regulation when they face appearance anxiety and weight-control pressure. It may therefore help clarify the indirect association between SC and eating disorders.

Physical exercise can be viewed as a behavioral resource related to individuals’ resource management. However, among Chinese female college students, this resource is likely to operate in a specific sociocultural context. With rapid Westernization, social media use, and increasing exposure to thin-ideal beauty standards, many young women face stronger pressure related to body shape, weight control, and appearance comparison. In this context, physical exercise may be closely connected with body management and self-discipline (Wong et al., 2023). Thus, physical exercise may function as an important boundary condition in the relationship between SC and eating disorders among Chinese female college students. Based on this reasoning, this study includes psychological resilience as a mediator and physical exercise as a moderator in the same model. It aims to further explain the resource-based pathway and boundary conditions linking SC and eating disorders in the sociocultural context of Chinese female college students.

### 1.1 Theoretical foundation

COR theory holds that people handle stress by obtaining, maintaining, and protecting resources (Hobfoll, 1989). COR theory conceptualizes stress as actual resource loss or the perceived threat of such loss. When individuals perceive ongoing resource depletion, limited replenishment, or blocked recovery, they tend to show negative psychological responses, for instance emotional exhaustion. In contrast, resource gains and recovery are associated with stronger coping capacity and relatively stable mental health. In addition, the resource caravan perspective within COR theory suggests that resources do not function in a vacuum. Rather, they often relate to, support, and accumulate alongside one another (Hobfoll, 1989; Mayer and Slone, 2024).

Within this framework, SC should not only be understood as a single internal psychological resource. It can also be viewed as a basic psychological resource within a resource caravan. According to Neff (2003) conceptualization, SC includes self-kindness, common humanity, and mindfulness. It shows how people handle themselves when they face suffering or personal inadequacy. Higher SC may help individuals respond to body-related pressure with less self-criticism and greater acceptance. In this way, it may be associated with lower emotional depletion and better preservation of psychological resources (Örnek et al., 2024). More importantly, SC may further support the development and mobilization of psychological resilience. Higher SC may be related to lower rumination and self-blame, as well as greater psychological space for recovery from negative experiences (Tingaz et al., 2022). As an adaptive resource, psychological resilience represents people’s ability to manage stress and restore psychological balance (Solmaz and Şimşek, 2025). Therefore, from the resource caravan perspective, SC and psychological resilience are not isolated from each other. Instead, they may reflect the connection and accumulation of internal resources, which may further explain the indirect association between SC and eating disorders (Robert et al., 2022; Tan, 2023).

Physical exercise also represents a behavioral resource within the framework of COR theory. On the one hand, it may be associated with less resource depletion related to body dissatisfaction, appearance anxiety, and eating-related rumination, as it is linked to stress release, emotion regulation, and attentional shift (Ye et al., 2022). On the other hand, it may also be related to resource recovery and accumulation through better physical function and more positive body experiences (Wong et al., 2021). Thus, physical exercise may serve both resource-protective and resource-gaining roles. It may also change the extent to which individuals rely on SC as an internal psychological resource, thereby moderating the association between SC and eating disorders (Huang et al., 2021). Therefore, under COR theory and the resource caravan perspective, including SC, psychological resilience, and physical exercise in the same model may help clarify how different types of resources are jointly related to the risk of eating disorders among female college students.

### 1.2 Self-compassion and eating disorders

The SC reflects a healthy stance toward oneself. It involves responding to one’s circumstances with kindness and nonjudgment, acknowledging that personal experiences are part of common humanity, and consciously accepting one’s shortcomings and imperfections (Sommerfeld and Shechory Bitton, 2020). Research reports a significant negative link between SC and eating disorders (Mousavi Asl et al., 2021). Örnek et al. (2024) stated that people with higher SC typically showed fewer eating disorder behaviors. SC may help individuals view emotional experiences and body image concerns more rationally. In turn, it may support more stable regulation of negative emotions related to body shape or weight and reduce reliance on extreme dietary control as a coping strategy (Yang et al., 2025). SC may also reduce self-criticism and promote acceptance of the body’s natural state. It may lessen the tendency to tie self-worth too closely to appearance and, in turn, relate to lower risk of eating disorders (Shaw and Cassidy, 2022). In addition, SC may relate to better emotion regulation, which may encourage healthier coping strategies rather than maladaptive eating behaviors to avoid stress or relieve tension (Batchelor et al., 2025). Therefore, SC may support efforts to prevent and address eating disorders.

### 1.3 The mediating role of psychological resilience

Psychological resilience denotes a person’s capacity to overcome challenges and bounce back from adverse circumstances (Li and Liu, 2025). Studies have revealed a strong negative link between psychological resilience and eating disorders (Robert et al., 2022). For example, using longitudinal data, Li et al. (2022) found that people with greater psychological resilience reported fewer eating disorder behaviors 12 months later. Psychological resilience may strengthen individuals’ confidence, which may make them more likely to use healthier coping strategies when facing body-image concerns or eating-related triggers, such as maintaining a balanced diet and engaging in moderate exercise. In turn, these strategies may relate to fewer extreme eating behaviors (Milligan et al., 2024). Psychological resilience may also foster healthier coping strategies. It may help individuals think more rationally and use effective strategies when they experience weight-related anxiety, which may relate to fewer eating disorders (McGarrity et al., 2022). Psychological resilience may also reduce excessive reliance on external evaluation. It may help individuals view their bodies and appearance more rationally and reduce extreme eating behaviors driven by others’ judgments or body-image dissatisfaction, which may relate to fewer eating disorder behaviors (Fergerson and Brausch, 2022). Therefore, psychological resilience may relate to a lower risk of eating disorders.

In addition, research has reported a significant positive link between SC and psychological resilience (Voon et al., 2021). For example, Ueno and Amemiya (2024) reported that people with higher SC also tended to report stronger psychological resilience. People with higher SC may regulate negative emotions more effectively. When they face body-related anxiety, they may respond to themselves with greater tolerance, adapt more successfully to challenges and difficulties, and show stronger psychological resilience (Wen et al., 2024). SC may also enhance self-acceptance. It may help individuals acknowledge their imperfections, reduce feelings of inferiority, and become better equipped to cope with diverse challenges, which may relate to higher psychological resilience (Tan, 2023). SC may also help individuals maintain a positive mindset after failures or setbacks and learn from these experiences, which may further relate to stronger psychological resilience (Liu et al., 2025). Individuals with higher psychological resilience typically report fewer eating disorder behaviors (Zhao et al., 2022). Previous research has primarily examined the link between SC and psychological resilience as well as the link between psychological resilience and eating disorders. However, research has not yet provided sufficient empirical evidence on whether psychological resilience mediates the link between SC and eating disorders. Accordingly, this study tests whether the link between SC and eating disorders is mediated by psychological resilience.

### 1.4 The moderating role of physical exercise

Physical exercise is one type of physical activity. It usually refers to repetitive, structured, and scheduled physical activity with the goal of preserving or enhancing health and physical fitness (Streetman et al., 2023). Huang et al. (2021) reported higher depression levels among individuals who showed both low physical exercise and low SC. For female college students, strong sociocultural emphasis on body image is often associated with heightened anxiety and stricter control over eating. In this context, physical exercise is associated with better mood, potentially through increased endorphin release, and it may also relate to fewer eating-disorder symptoms that stem from appearance- and weight-related concerns (Wong et al., 2023). In addition, physical exercise can enhance physical fitness and health, and it may promote more positive perceptions of one’s body. By supporting SC, physical exercise may help individuals view their body image in a more tolerant and healthy way, reduce excessive control and anxiety about eating and body shape, and relate to a lower risk of eating disorders (Wong et al., 2021). People often use physical exercise to regulate emotions. It may help individuals release stress and negative emotions, shift attention away from eating-related rumination and appearance concerns, and relate to fewer eating disorders (Ye et al., 2022; Zhao et al., 2024). Accordingly, physical exercise may moderate the link between SC and eating disorders.

### 1.5 The present study

**Figure 1:**
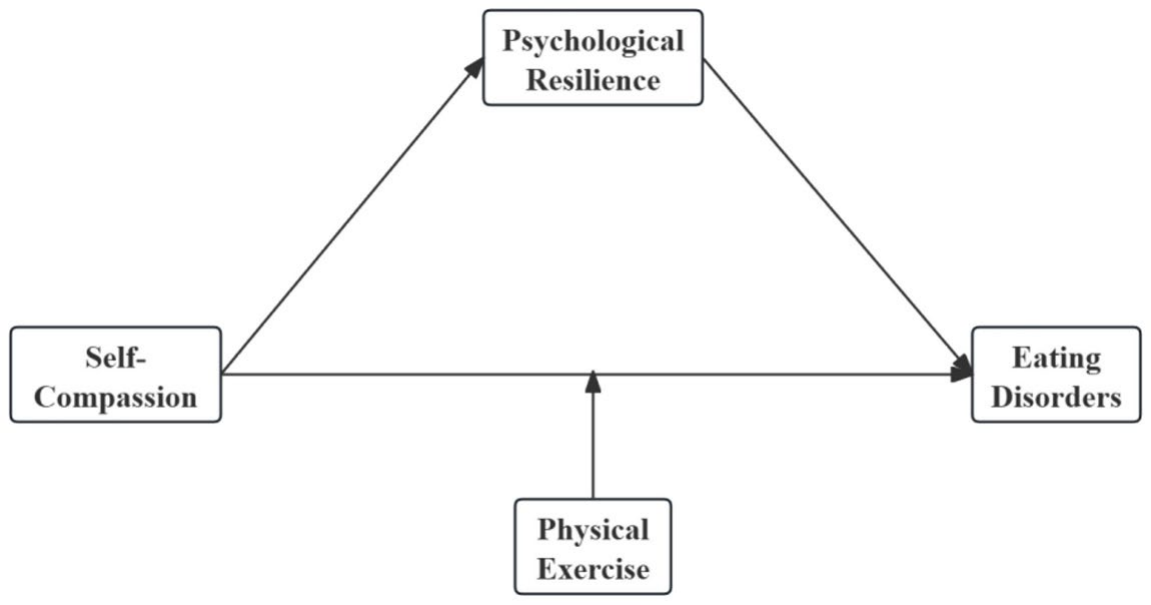
Conceptual model.

Although prior work links SC to eating disorders, the specific mechanisms involved, especially among female college students, remain insufficiently understood. No study has clearly tested whether psychological resilience accounts for the link between SC and eating disorders among female college students, and the literature also offers limited evidence on whether physical exercise moderates this link. Therefore, guided by COR theory, we developed a moderated mediation model to examine whether SC relates to eating disorders through psychological resilience and whether physical exercise moderates these associations. This study seeks to clarify the mechanisms linked to eating disorders among female college students and to offer a theoretical rationale for psychological interventions targeting eating disorders. Figure 1 displays the conceptual model. Based on this framework, we propose the following hypotheses:

*H1*: SC is negatively linked to eating disorders.

*H2*: SC is positively linked to psychological resilience.

*H3*: Psychological resilience is negatively linked to eating disorders.

*H4*: Psychological resilience mediates the link between SC and eating disorders.

*H5*: Physical exercise moderates the link between SC and eating disorders.

## 2 Methods

### 2.1 Participants and procedure

To improve sample representativeness and reduce selection bias, we used simple random sampling among female college students from three universities in China between October and November in 2025. Each eligible student had an equal probability of being selected. Participants filled out the online survey on the Wenjuanxing platform. Before responding, all participants read and provided informed consent. We followed confidentiality principles throughout the study, and we used the data only for research purposes. Using G*Power 3.1, we performed an *a priori* power analysis to ascertain an acceptable sample size (Faul et al., 2009). We used eight predictors, a medium effect size of *f*^2^ = 0.15, *α* = 0.05, and 1 − *β* = 0.95 in the multiple linear regression model. The analysis indicated that the minimum required sample size was *N* = 160. We collected 928 questionnaires. After excluding 52 invalid questionnaires (more than 20% missing responses or selecting “strongly agree/strongly disagree” for over 80% of the questionnaire statements), we retained 876 legitimate surveys, resulting in a 94.40% valid response rate. Sophomores accounted for the largest proportion of the sample (27.1%). Table 1 reports additional demographic characteristics.

**Table 1:** Demographic characteristics.

Variable	Category	*n*	Percentage (%)	*p*-value
Age	18–20 years	388	44.3	0.092
	21–22 years	474	54.1	
	≥23 years	14	1.6	
Grade	Freshman	230	26.3	0.338
	Sophomore	237	27.1	
	Junior	185	21.1	
	Senior	224	25.5	
Registered residence	Rural	603	68.8	0.123
	Urban	273	31.2	
Only child	Yes	122	13.9	0.416
	No	754	86.1	

### 2.2 Measures

#### 2.2.1 Self-compassion

We measured SC using the self-compassion scale revised by Chen et al. (2011) for female college students. Three categories are covered by the 12-item scale: self-kindness, mindfulness, and common humanity. Although these three dimensions represent different aspects of SC, this study concentrated on the overall level of SC rather than the distinct role of each dimension. Therefore, we used the 12-item mean score as a composite indicator. Participants used a 5-point response format, and higher scores indicated greater SC. Prior research has reported good reliability and validity for this scale in Chinese samples (Wang S. et al., 2023). In this study, we used the mean score of this scale for analysis, and the Cronbach’s alpha for the entire SC scale was 0.915.

#### 2.2.2 Psychological resilience

We assessed psychological resilience with the 10-item Connor–Davidson resilience scale revised by Connor and Davidson (2003) for female college students. This scale follows a single-factor structure and uses a 5-point response format, where higher scores reflect higher psychological resilience. Studies have shown strong reliability and validity for this scale in Chinese samples (Mei et al., 2022). In this study, we used the mean score of this scale for analysis, and the Cronbach’s alpha for the total psychological resilience scale was 0.907.

#### 2.2.3 Physical exercise

We assessed physical exercise with the physical exercise questionnaire revised by Liang (1994) for female college students. The instrument includes three items that assess exercise intensity, frequency, and duration. It uses a 5-point response format. According to the original scoring method of this scale, the overall physical exercise score was determined as follows: exercise intensity × (exercise duration − 1) × exercise frequency. Here, subtracting 1 from exercise duration is part of the original scoring formula, because the exercise duration item uses categorical codes from 1 to 5. This treatment converts the duration dimension into a range from 0 to 4, which helps avoid overestimating very short or minimum levels of exercise duration. Higher scores indicate a higher overall level of physical exercise. Prior research has validated this instrument in Chinese samples (Zhang et al., 2025). Because this instrument uses exercise intensity, duration, and frequency to form a composite behavioral index, rather than multiple homogeneous items to form a latent scale, we did not calculate Cronbach’s alpha in this study.

#### 2.2.4 Eating disorders

We using the eating attitudes test-26 introduced by Garner et al. (1982) to evaluate eating disorders among female college students. The scale contains 26 items across three domains: dieting, bulimia, and oral control, and it uses a 6-point response format. Following the recommended screening scoring procedure, we recoded responses as follows: 3 = always, 2 = usually, 1 = often, and 0 = sometimes/rarely/never. Total scores spanned 0–78, with higher scores indicating more pronounced eating-disorder symptoms. Studies have validated this scale in Chinese samples (Wang J. et al., 2023). In this study, we used the total EAT-26 score for analysis, and the Cronbach’s alpha for the total scale was 0.962.

### 2.3 Data analysis

The theoretical model and research hypotheses guided the data analysis strategy. We conducted the analysis in three steps: preliminary tests, measurement model testing, and hypothesis testing. First, we used SPSS to conduct descriptive statistics, skewness and kurtosis tests, and Pearson correlation analysis to examine variable distributions and preliminary correlations. Because this study collected cross-sectional data through self-report questionnaires, we further used Harman’s single-factor test and the latent method factor approach to assess common method bias (CMB). Second, we used AMOS to conduct confirmatory factor analysis (CFA) (Byrne, 2016) to assess the fit of the measurement model for the main variables. We also examined the reliability and validity of the scales using Cronbach’s alpha, composite reliability, average variance extracted (AVE), and the HTMT ratio. Before testing the core model, we checked multicollinearity using tolerance and variance inflation factor values.

Finally, we used the SPSS PROCESS macro 4.0 to conduct regression-based conditional process analysis (Hayes, 2022). Specifically, Model 4 tested the indirect role of psychological resilience. Model 5 further tested the moderating role of physical exercise while retaining the indirect pathway through psychological resilience. We tested the significance of the indirect effect using 5,000 bootstrap resamples. We regarded the indirect effect as statistically significant when the 95% confidence interval did not include 0 (Preacher and Hayes, 2008). Given the cross-sectional design of this study, all results should be interpreted only as statistical indirect and conditional associations, rather than as causal evidence.

## 3 Results

### 3.1 Descriptive statistics and correlation analysis

The correlation results showed that SC was positively correlated with psychological resilience (*r* = 0.666, *p* < 0.001) and negatively correlated with eating disorders (*r* = −0.675, *p* < 0.001). Psychological resilience was also negatively correlated with eating disorders (*r* = −0.671, *p* < 0.001) (Table 2).

**Table 2:** Descriptive statistics and correlations.

Variable	*M* ± SD	Sk	Kur	SC	PR	PE	EDs
SC	3.018 ± 0.790	−0.233	0.160	1			
PR	3.214 ± 0.775	−0.305	0.948	0.666***	1		
PE	24.270 ± 21.871	1.346	1.633	0.457***	0.404***	1	
EDs	24.822 ± 21.438	0.922	−0.477	−0.675***	−0.671***	−0.318***	1

SC, self-compassion; PR, psychological resilience; PE, physical exercise; Eds, eating disorders; ***p < 0.001.

### 3.2 CMB

Because this study collected cross-sectional data using questionnaires, we tested for CMB. Six factors with eigenvalues greater than 1 were first identified by Harman’s single-factor test, and the first component accounted for 39.983% of the variance. This figure was less than the widely accepted 40% cutoff (Podsakoff and Organ, 1986). In addition, we employed the latent method factor technique (Podsakoff et al., 2003). The chi-square difference test showed no significant difference between baseline model and measurement model (Δχ^2^ = 1.956, Δdf = 1, *p* > 0.05). These findings suggest that CMB probably did not provide a significant risk.

### 3.3 Measurement model testing

Before testing the hypotheses, we first examined the fit, reliability, and validity of the measurement model. The entire measurement model suited the data well, according to the CFA: CMIN/DF = 2.175, RMSEA = 0.037, CFI = 0.947, IFI = 0.947, and TLI = 0.944. The measuring methodology demonstrated good construct validity since all values satisfied the suggested criteria.

Further reliability and validity analyses showed that the Cronbach’s alpha for all variables ranged from 0.907 to 0.962, and the composite reliability ranged from 0.923 to 0.965. The scales demonstrated strong internal consistency, since all of the values were over 0.70. The AVE ranged from 0.511 to 0.546, all above 0.50, indicating acceptable convergent validity for each latent variable. The HTMT ranged from 0.717 to 0.728, all below 0.90, indicating good discriminant validity among the latent variables.

### 3.4 Collinearity diagnostics

To guarantee model estimation stability, we further tested multicollinearity among the predictors. The tolerance values for all variables ranged from 0.514 to 0.773, all above 0.10. The VIF values ranged from 1.293 to 1.946, all below the recommended criterion of 3.3. These results suggest that the model did not show serious multicollinearity.

### 3.5 Mediation analysis

We used Model 4 of the IBM SPSS PROCESS macro to examine whether psychological resilience was statistically involved in the indirect association between SC and eating disorders. The regression results are shown in Table 3. After we included psychological resilience in the model, SC remained significantly and negatively associated with eating disorders (*β* = −11.130, *t* = −13.379, *p* < 0.001), and psychological resilience was also strongly and inversely correlated with eating disorders (*β* = −11.004, *t* = −12.978, *p* < 0.001). In addition, there was a strong positive link between SC and psychological resilience (*β* = 0.654, *t* = 26.424, *p* < 0.001).

**Table 3:** Hierarchical regression results for the mediation model.

Outcome variable	Predictor variable	*R*	*R* ^2^	*F*	*β*	*SE*	*t*
PR	SC	0.666	0.444	698.218	0.654	0.025	26.424***
EDs	SC	0.738	0.544	520.645	−11.130	0.832	−13.379***
	PR				−11.004	0.848	−12.978***

***p < 0.001.

The bootstrap results showed a significant indirect link between SC and eating disorders through psychological resilience. The indirect effect linking SC to eating disorders through psychological resilience was −7.195, and the 95% bootstrap confidence interval was [−8.671, −5.917], which did not include 0 (Table 4). This result indicates a significant indirect effect. At the same time, the direct effect between SC and eating disorders remained significant, suggesting that psychological resilience was consistent with a partial mediation pattern. Given the cross-sectional design of this study, this result should be understood as a statistical indirect association rather than evidence of a causal mediation process.

**Table 4:** Bootstrap analysis of the mediation effect among SC, psychological resilience, and eating disorders.

Effect	Path	Effect value	*SE*	Bootstrap 95% CI	
				LLCI	ULCI
Total effect	SC → EDs	−18.324	0.677	−19.653	−16.996
Direct effect	SC → EDs	−11.130	0.832	−12.763	−9.497
Indirect effect	SC → PR → EDs	−7.195	0.706	−8.671	−5.917

### 3.6 Moderation analysis

To examine whether physical exercise moderated the link between SC and eating disorders, we further created an interaction term for the moderation analysis. The results showed that before adding the interaction term, the model accounted for 54.4% of the variance in eating disorders (*R*^2^ = 0.544). After we included the interaction between SC and physical exercise, *R*^2^ increased to 0.577, which reflects an additional 3.3% of explained variance. This result suggests that the moderation term contributed incremental explanatory power to the model. Further analyses showed that SC was significantly and negatively associated with eating disorders (*β* = −17.696, *t* = −15.572, *p* < 0.001). The interaction term was also significant (*β* = 0.230, *t* = 8.048, *p* < 0.001), indicating that physical exercise significantly moderated the association between SC and eating disorders (Table 5).

**Table 5:** Moderation analysis.

Path	*β*	*SE*	*t*	Bootstrap 95% CI		*p*
				LLCI	ULCI	
SC → EDs	−17.696	1.136	−15.572	−19.926	−15.466	<0.001
PE × SC → EDs	0.230	0.029	8.048	0.174	0.286	<0.001

## 4 Discussion

This study showed a significant negative association between SC and eating disorders, which supported H1. This finding aligns with Mousavi Asl et al. (2021), suggesting that higher SC tends to coincide with fewer eating disorder behaviors. SC emphasizes responding to painful experiences with self-kindness, common humanity, and mindfulness. When female college students face body dissatisfaction, weight anxiety, or loss of control over eating, higher SC may be associated with a lower tendency to view these experiences as personal failures and a lower likelihood of falling into shame, self-blame, and harsh self-criticism (Örnek et al., 2024). Existing research shows that SC can attenuate the associations of body dissatisfaction and perfectionism with eating disorders (Bergunde and Dritschel, 2020). Therefore, individuals with higher SC may be less likely to regard a perfect body or strict dietary control as an important source of self-worth, and may show lower levels of eating disorders related to body dissatisfaction and perfectionistic standards (Batchelor et al., 2025). SC may also buffer the salience of appearance-based comparisons and social evaluation. When individuals face appearance-related pressure, SC may help them maintain a stronger sense of self-worth and reduce attempts to gain reassurance by tightly controlling body weight, which is associated with fewer eating disorders (Shaw and Cassidy, 2022). At the same time, after dietary deviation or temporary loss of control, SC may also be associated with a lower likelihood of entering the failure self-blame compensatory control cycle and with a greater tendency to return to a relatively regular eating pattern (Yang et al., 2025). Overall, SC relates to fewer eating disorder behaviors among female college students.

Psychological resilience served as a mediator in the association between SC and eating disorders, supporting H2, H3, and H4. This result is consistent with Tan (2023), suggesting that SC relates to higher psychological resilience, and psychological resilience relates to fewer eating disorder behaviors (Li et al., 2022). SC may represent an important internal psychological resource for female college students, supporting their capacity to recover in stressful situations. Higher psychological resilience, in turn, is associated with fewer eating disorder behaviors (Liu et al., 2025; McGarrity et al., 2022). Specifically, female college students with higher SC may be more likely to accept personal imperfections when facing body dissatisfaction or pressure from external evaluation. This tendency may reduce the emotional drain associated with self-criticism and shame and support recovery under stress, which is reflected in stronger psychological resilience (Wen et al., 2024). Female college students with stronger psychological resilience may accept negative emotions more readily, maintain stable daily routines under stress, and use more flexible regulation strategies. These patterns are associated with fewer eating disorder behaviors in the context of appearance-related pressure (Milligan et al., 2024). Therefore, SC may relate to lower eating disorder behaviors among female college students indirectly through higher psychological resilience. It is worth noting that psychological resilience showed a partial mediation pattern in this study. This suggests that the association between SC and eating disorders cannot be fully explained by psychological resilience. Beyond psychological resilience, factors such as self-esteem, social support, body appreciation, appearance comparison, and perfectionism may also be involved in this process. Future research may include these variables to provide a more complete explanation of the multiple psychological mechanisms linking SC and eating disorders.

Physical exercise moderated the association between SC and eating disorders, supporting H5. Notably, the interaction term was positive, indicating that physical exercise weakened the negative association between SC and eating disorders. From a resource-substitution perspective within COR theory, SC represents an internal psychological resource and may be associated with less self-criticism and greater acceptance when individuals face body-related pressure. Physical exercise, in contrast, represents a behavioral resource. It may provide another source of resources for coping with body- and eating-related pressure through stress release, emotion regulation, and positive body experiences (Hobfoll, 1989). When physical exercise is high, individuals may already have sufficient behavioral regulation resources, so the additional psychological resource advantage linked to SC may become relatively weaker (Solmaz et al., 2025). In contrast, when physical exercise is low, behavioral regulation resources may be insufficient, and SC may become a more central psychological protective resource (Wong et al., 2023). This explanation does not contradict the resource caravan principle in COR theory. The resource caravan principle emphasizes that resources tend to accumulate and support one another. However, resource accumulation does not mean that different resources will always strengthen each other’s association with the same outcome variable. Therefore, the findings of this study are better understood as differences in marginal effects under conditions of sufficient resources. In other words, physical exercise may play a resource-substitution moderating role, rather than simply strengthening the association between SC and eating disorders.

## 5 Implications and limitations

### 5.1 Theoretical implications

Drawing on COR theory, this study offers a more fine-grained account of the resource-based mechanisms underlying eating disorder symptoms among female college students. First, the study conceptualized SC as a key internal psychological resource. In high-depletion contexts such as body image pressure and setbacks in eating control, higher SC tends to co-occur with lower self-criticism and weaker negative emotional reactions, and it relates to lower levels of eating disorder symptoms. This pattern provides a more direct resource-based explanation for individual differences in eating disorder symptoms from a resource-loss perspective. Second, the mediating role of psychological resilience highlights a resource-linking mechanism. SC may operate as an upstream resource that relates to stronger psychological resilience, and psychological resilience represents a relatively stable, readily mobilized adaptive resource that relates to lower eating-disorder symptoms. This pattern reflects the resource accumulation and resource caravans emphasized in COR theory and moves the role of SC beyond a simple correlation toward a testable resource pathway. Third, the moderation results for physical exercise in the SC–eating-disorder link suggest that resource effects vary across contexts. This pattern aligns with COR theory views on resource substitution and compensation. Physical exercise can be viewed as a behavioral pathway for resource gain. When exercise levels are higher, individuals may obtain additional recovery and regulation resources through exercise, which may make the unique association between SC and eating disorder symptoms relatively weaker. In contrast, when exercise levels are lower and resource replenishment is limited, the protective association of SC may become more salient. Overall, this study integrated internal resources (SC), adaptive resources (psychological resilience), and behavioral resources (physical exercise) within a single model. By doing so, it extends the theoretical applicability of COR theory in the eating disorders literature by clarifying resource transmission, resource compensation, and their conditional associations.

### 5.2 Practical implications

This study provides actionable insights for health promotion and eating disorder risk management in universities. First, the findings identify SC as a key entry point for campus mental health services. Universities can integrate SC training into counseling, group programs, and mental health courses, such as reducing self-criticism and strengthening self-acceptance and emotional awareness. These efforts may provide more targeted support for high-depletion situations such as appearance pressure and setbacks in eating control, and they may relate to lower levels of eating disorders. Second, the mediating role of psychological resilience suggests that interventions should go beyond attitudinal or cognitive adjustment and also strengthen trainable adaptive capacities. These capacities include coping with stress, emotion regulation, recovery from setbacks, and problem solving. By enhancing students’ stability and recovery in triggering situations, universities may build a more sustainable support pathway. In addition, universities may combine exercise contexts with the cultivation of psychological resources. For example, counseling centers may work with physical education departments to offer SC-oriented yoga courses, mindful walking groups, low-intensity group exercise, or body awareness training. In these activities, instructors may guide students to focus on body function, breathing rhythm, emotional changes, and recovery experiences after exercise, rather than placing too much emphasis on weight control or exercise performance. Finally, universities may strengthen coordination among mental health screening, physical education courses, psychological counseling, and referral services in university hospitals. This can help them identify students with body dissatisfaction, extreme dieting, excessive exercise, or risk of eating disorders earlier and provide more targeted support.

### 5.3 Limitations and future directions

This study has several limitations, which also identifies directions for future research.

First, we measured all core variables at a single time point using self-report questionnaires. Although this study built the mediation model on existing theory and empirical research, the cross-sectional design cannot determine the temporal order among SC, psychological resilience, physical exercise, and eating disorders. Therefore, the findings of this study can only be interpreted as statistical associations and should not be treated as evidence of causal processes. Future research should not be limited to longitudinal designs. Researchers may also conduct experimental or intervention studies to examine the role of SC in the risk of eating disorders more directly. For example, researchers could use randomized controlled trials to compare changes in body dissatisfaction, self-criticism, psychological resilience, and eating disorder symptoms between a SC training group and a control group. They could also further examine whether psychological resilience is involved in the intervention effects.

Second, self-report data may reflect social desirability and recall bias. This concern may be particularly salient for sensitive behaviors such as eating disorders and physical exercise, where underreporting or strategic responding may occur. Future studies can incorporate multi-informant data and more objective indicators, such as wearable- or platform-based activity records, behavioral indicators related to weight management, and clinical screening tools or semi-structured interviews, to strengthen measurement robustness and ecological validity.

Third, the sample consisted of Chinese female college students, so caution is needed when generalizing the findings. Female college students are indeed one of the groups with a relatively high risk of eating disorders, but eating disorder symptoms may vary across gender groups. For example, the risk of eating disorders among men may not mainly involve the pursuit of thinness or weight control. Instead, it may be more closely reflected in muscle dysmorphia, muscle-oriented eating, excessive exercise, or dissatisfaction with muscularity. Therefore, the relationships among SC, psychological resilience, physical exercise, and eating disorders found in this study cannot be directly generalized to male college students or other groups. Future research may further examine this model among male college students, adolescents, non-student groups, and broader community samples. It may also compare whether the relevant pathways differ across gender and cultural backgrounds.

Fourth, this study mainly drew on COR theory and examined the statistical indirect association between SC and eating disorders through psychological resilience. However, it did not include more specific body-related cognitive variables, such as body image flexibility and body appreciation. Body appreciation reflects individuals’ acceptance, respect, and positive evaluation of their own bodies. Body image flexibility emphasizes whether individuals can accept body-related experiences and maintain value-consistent behavioral choices when they face body dissatisfaction or negative body evaluations. Compared with psychological resilience, these two variables focus more directly on how individuals evaluate, accept, and respond to their own body experiences. They may represent important body-specific mechanisms linking SC and eating behaviors. Future research may further include body appreciation and body image flexibility as mediators in the model, and compare their relative roles with variables such as psychological resilience, body dissatisfaction, self-esteem, social support, and perfectionism.

## 6 Conclusion

Using data from 876 Chinese female college students, this study tested a moderated mediation model. The results indicated a significant negative association between SC and eating disorders, and psychological resilience served as a significant mediator in this association. Further analyses indicated that physical exercise significantly moderated the direct link between SC and eating disorders. As physical exercise increased, the negative association between the two became weaker. From the perspective of COR theory, this study suggests that SC can be understood as an internal psychological resource, psychological resilience reflects an adaptive resource in stressful contexts, and physical exercise represents a behavioral resource. Together, these three resources suggest that managing the risk of eating disorders should not focus only on a single psychological factor. Instead, it should adopt a more holistic perspective that considers both psychological resources and physical or behavioral resources. At the practical level, universities may combine SC training, psychological resilience development, and health-oriented physical exercise support to provide students with more comprehensive programs for eating disorder risk prevention and mental health promotion. It should be noted that this study used cross-sectional self-report data, so the findings are better interpreted as patterns of association among variables rather than as causal relationships. Future research may use experimental, longitudinal, or multi-source designs and include samples from different gender and cultural groups to further examine the applicability and boundary conditions of this model.

## Data Availability

The datasets presented in this study can be found in online repositories. The names of the repository/repositories and accession number(s) can be found in the article/supplementary material.
